# Pathological changes in the lymphatic system of patients with secondary upper limb lymphoedema

**DOI:** 10.1038/s41598-019-44735-w

**Published:** 2019-06-11

**Authors:** Taro Mikami, Asumi Koyama, Koukichi Hashimoto, Jiro Maegawa, Yuichiro Yabuki, Shintaro Kagimoto, Shinya Kitayama, Tomohiro Kaneta, Kazunori Yasumura, Shinobu Matsubara, Toshinori Iwai

**Affiliations:** 1 Department of Plastic and Reconstructive Surgery, Yokohama City University, School of Medicine, Yokohama, Japan; 2 Tokyo Medical Center, Tokyo, Japan; 3 Department of Plastic and Reconstructive Surgery, Yokohama City University, Graduate School of Medicine, Yokohama, Japan; 4 Department of Radiology, Yokohama City University, School of Medicine, Yokohama, Japan; 5 Department of Plastic and Reconstructive Surgery, Kanagawa Children’s Medical Center, Yokohama, Japan; 6 Department of Vascular Surgery, Yokohama Minami Kyousai Hospital, Yokohama, Japan; 7 Department of Maxillofacial Surgery, Yokohama City University, School of Medicine, Yokohama, Japan

**Keywords:** Physiology, Anatomy, Medical research, Chronic inflammation

## Abstract

Secondary upper limb lymphoedema is usually caused by lymphatic system dysfunction. Diagnosis is primarily based on clinical features. However, there are no distinct diagnostic criteria for lymphoedema. Although conventional lymphoscintigraphy is a useful technique to diagnose the severity of lymphoedema, the resultant data are two-dimensional. In this study, we examined the pathology of lymphoedema using single photon emission computed tomography-computed tomography lymphoscintigraphy (SPECT-CT LSG), a new technique that provides 3-dimensional information on lymph flow. We observed lymph flow pathways in the subcutaneous and muscle layers of the upper limbs. A significant positive correlation was found between the dermal back flow (DBF) type and the visualization of lymph nodes around the clavicle (*p* = 0.000266), the type of lymph flow pathways and the visualization of lymph nodes around the clavicle (*p* = 0.00963), and the DBF type and the lymph flow pathway (p = 0.00766). As the severity of lymphoedema increased, the DBF appeared more distally in the upper limb and the flow into the lymph nodes around the clavicle decreased, whereas the lymph flow pathways in the muscle layer became dominant. These findings demonstrate the features of lymphoedema pathology and the functional anatomy and physiology of the lymphatic system without the need for cadaver dissection.

## Introduction

Lymphoedema is caused by dysfunction of the lymphatic system that leads to pathological retention of fluid and solutes^1,2^. Patients suffer both physically and mentally during the clinical course of the disease, and the economic burden is not negligible^1,3,4^.

Lymphoedema is classified into primary and secondary types, on the basis of the cause and presence of underlying disease. Primary lymphoedema is congenital or of unknown origin, whereas secondary lymphoedema is caused by infection, trauma, or cancer treatment^2,5,6^. In developed countries, breast cancer typically precedes the onset of secondary upper limb lymphoedema. According to a meta-analysis by DiSipio *et al*. in 2013, 21.4% of breast cancer patients have upper limb lymphoedema, 28.2% of patients who undergo axillary lymph node dissection develop upper limb lymphoedema, and 5.6% of patients who undergo sentinel lymph node biopsy develop upper limb lymphoedema^7^.

Although clinical history and physical examination are important in the diagnosis of lymphoedema, there are no distinct diagnostic criteria. Some modalities, such as lymphoscintigraphy (LSG), which is recommended by the International Society of Lymphology, and near-infrared fluorescent lymphography with indocyanine green, are considered effective for the diagnosis of lymphoedema^8,9^. A correlation has previously been reported between the clinical stage and the types of images acquired by LSG in patients with secondary upper limb lymphoedema^10^. Dermal back flow (DBF) on LSG images refers to the phenomenon of lymph back flow from the collecting lymph duct to the dermis^11,12^. Conditions affecting the collecting lymph duct tend to deteriorate from the proximal region; hence, the lymphoedema becomes increasingly severe as the DBF appears and progresses distally^13^. Conversely, the number of patients in whom lymph nodes around the clavicle can be identified by LSG decreases as the severity increases^10^. Although a correlation was suspected between the location of DBF, severity of lymphoedema, and the positive rate of the lymph nodes around the clavicle in an LSG study in 2011, the underlying mechanism has not been investigated in previous research^10^.

Recently, we have used single photon emission computed tomography-computed tomography (SPECT-CT) LSG for the diagnosis of lymphoedema. This modality provides three-dimensional live images of lymph flow, unlike cadaveric studies that provide only anatomical information on the lymphatic system^14^. In addition, it is possible to assemble data from SPECT-CT LSG in the form of images, similar to images obtained via plain LSG.

The aim of this study was to investigate the association between DBF patterns, the lymph nodes around the clavicle and the lymph flow of the patients with secondary upper limb lymphoedema using the images from SPECT-CT LSG.

## Results

### Patient characteristics

Of the 101 patients included in the study, 100 had lymphoedema secondary to malignancies, except for one patient who had suffered a traumatic fracture of the clavicle on the affected side. Among the 100 patients who had malignancies, 97 had breast cancer, one patient had Merkel cell carcinoma, one patient had a sarcoma in the chest and one patient had malignant lymphoma (Table 1). The treatments for the primary diseases varied widely, including axillary lymph node dissection, radiation, chemotherapy and combinations of these (Table 2).

**Table 1:** Primary diseases of the patients Both limbs were involved in 3 patients.

Primary disease	Number of cases
Breast cancer	97
Merkel cell carcinoma	1
Sarcoma in the chest	1
Malignant lymphoma	1
Fracture of the clavicle	1
*Total*	101

**Table 2:** Details of the therapeutic options of the patients. One patient did not undergo surgery.

		axillary dissection	
		+	−
radiation	+	48/6	0/2
	−	33/11	0/1

(number/number: chemotherapy +/−).

### Assessment of the non-affected side

**Figure 1:**
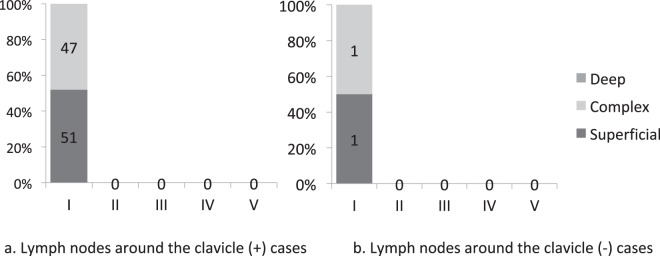
Association between the type of DBF pattern and the lymph flow pathway in the unaffected limbs. (**a**) Cases in which the lymph nodes around the clavicle were positive. In total, 51 cases out of 98 had a ‘superficial dominant’ type of lymph flow, while the others had a ‘complex’ type of lymph flow. (**b**) Cases in which the lymph nodes around the clavicle were negative. Lymph nodes around the clavicle were not observed in two cases on the single photon emission computed tomography-computed tomography lymphoscintigraphy (SPECT-CT LSG) images in the unaffected upper limb. The superficial pathway was dominant in one case, whereas the deep pathways were as active as the superficial pathways in the other case. None of the cases were classified as having ‘deep dominant’ lymph flow.

There were 98 non-affected limbs because there were 3 cases of bilateral upper limb lymphoedema. In total, 96 out of 98 limbs were positive for visualized lymph nodes around the clavicle (LNP group). In the LNP group, 52 cases were categorized into the superficial dominant lymph flow group and none were categorized into the deep dominant group. All cases in the LNP group demonstrated a type I pattern of DBF (Fig. 1a).

No lymph nodes were present around the clavicle on the non-affected side in two cases. The lymph flow pathway was classified as superficial dominant in one case, and the other was classified as the complex type. Both were classified under the type I pattern of DBF (Fig. 1b). There were no split decisions between the two referees.

### Association between the types of DBF and the visualization of lymph nodes around the clavicle on the affected side

**Figure 2:**
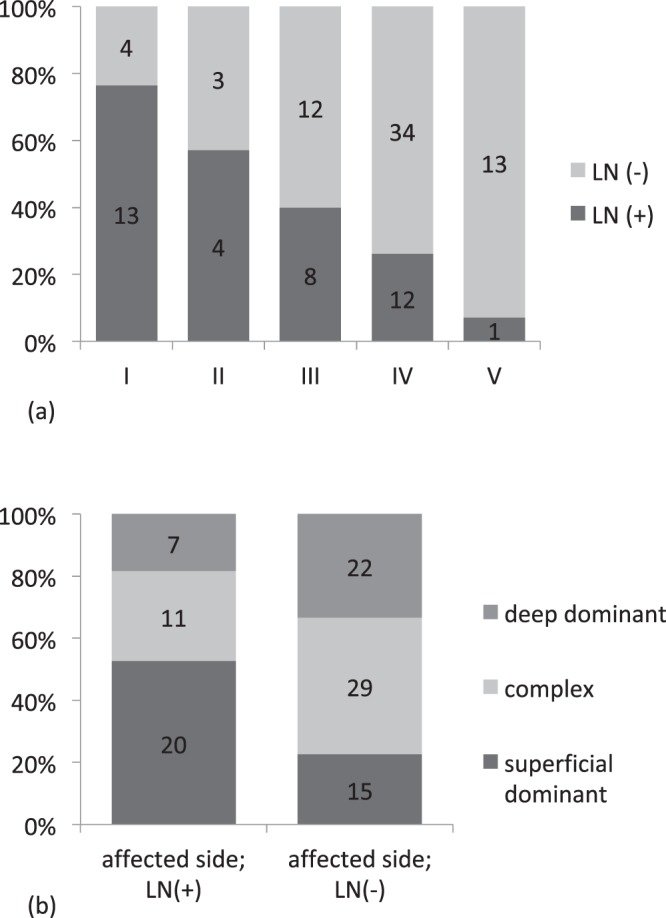
Association between the types of dermal back flow and the visualization of lymph nodes around the clavicle in the affected side. (**a**) Association between the type of lymph flow pathways and the visualization of the lymph nodes around the clavicle in the affected limb. The lymph nodes around the clavicle become obscure, as the dermal back flow appears in the distal site. (**b**) Association between the visualization of lymph nodes around the clavicle in the affected side and the type of lymph flow pathways. Fisher’s exact test found a statistically significant association (*p* = 0.00963; <0.01).

A positive correlation was found between the type of DBF and the visualization of lymph nodes around the clavicle on the affected side, which was statistically significant (*p* = 0.000266; <0.01; Table 3, Fig. 2a), and this was similar to the findings of our previous report^10^. As the DBF appears in a more distal part of the upper arm, the lymph nodes around the clavicle are less clearly visualized.

**Table 3:** Relation between the dermal back flow type and the visualization of lymph nodes around the clavicle. Significant relevance was noted (Fisher’s exact test, *p* = 0.000266; <0.01).

Type	LN (+)	LN (−)
I	13	4
II	4	3
III	8	12
IV	12	34
V	1	13

LN (+): lymph nodes around the clavicle were observed; LN (−): no lymph nodes around the clavicle were observed.

### Lymph flow pathway and lymph nodes around the clavicle

A statistically significant association was observed between the type of lymph flow pathway and visualization of lymph nodes around the clavicle on the affected side (*p* = 0.00963; <0.01; Fig. 2b). With respect to the presence of lymph nodes around the clavicle, 50% were superficial pathway-dominant and less than 20% were deep pathway-dominant.

### DBF type and lymph flow pathway

**Figure 3:**
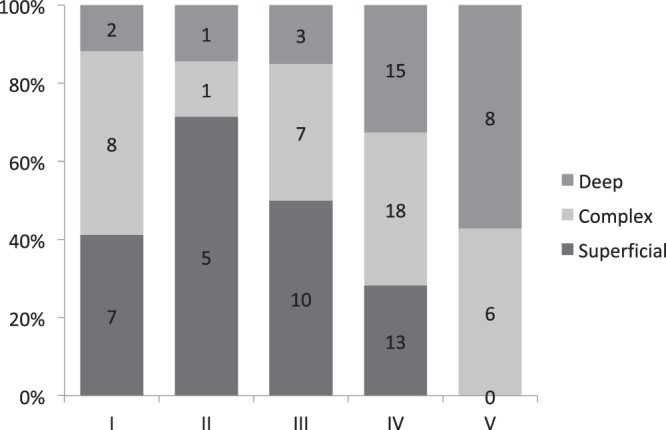
Association between the classification of dermal back flow type and lymph flow pathway in the affected limbs regardless of the lymph nodes around the clavicle. The deep pathways seem to be activated as dermal back flow appears in the distal site. A statistically significant association was observed between these two factors (Fisher’s exact test, *p* = 0.00963).

**Figure 4:**
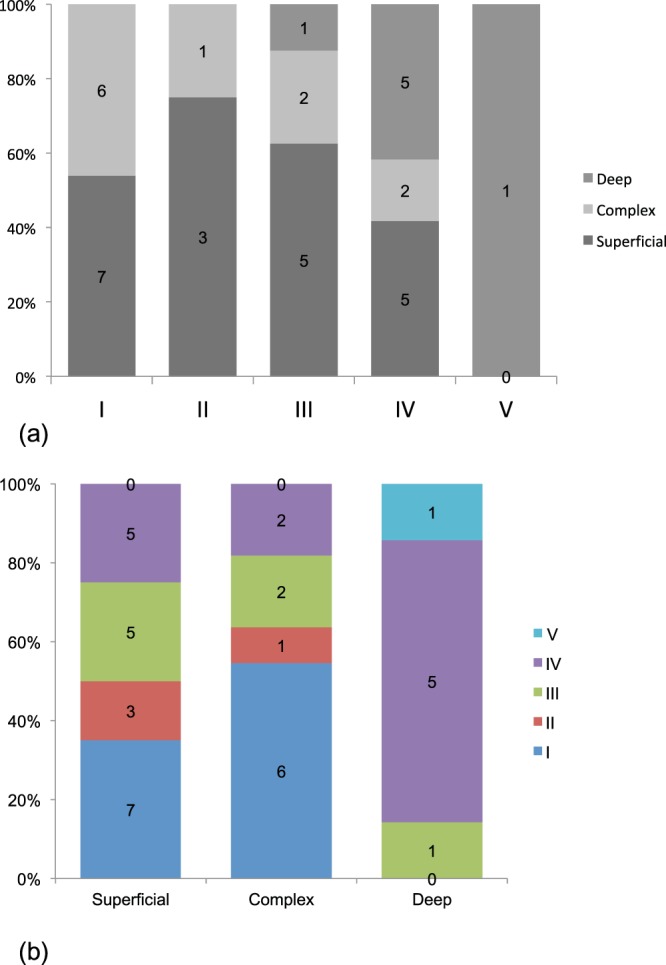
Association between the classification of dermal back flow type and lymph flow pathways in lymph node-positive cases of the affected limbs. (**a**) The superficial pathways seem to be inferior; dermal back flow appears in the distal site, although only one case was classified as type V. (**b**) Superficial pathways are active and superior in cases with a type I, II or, III dermal back flow pattern based on the findings of the ‘Superficial’ and ‘Complex’ columns in this chart.

A consensus on the assessment of lymph flow pathways in the affected limb could not be reached in 2 cases of type V DBF, and the final decision was deep dominant in both after discussion (Table 4, Fig. 3). A statistically significant association was observed between the DBF type and the lymph flow pathway (p = 0.00766; <0.01). In other words, the deep pathway-dominant types increased as the DBF appeared in a more distal part of upper limb regardless of the lymph nodes around the clavicle. Moreover, as the severity of the DBF type increased from type II to type V, the number of superficial dominant types decreased. With respect to the presence of lymph nodes around the clavicle, the lymph flow pattern of type I DBF was similar to that of the non-affected side (*p* = 1.00; Figs 1a, 4a). There were no deep pathway-dominant types, and the number of superficial pathway-dominant types was similar to the number of complex pathway types.

**Table 4:** Relationship between the types of dermal back flow and the lymph flow pathway in the cases with visualized lymph nodes around the clavicle. Statistical relevance was observed between the dermal back flow type and the lymph flow pathway (Fisher’s exact test, *p* = 0.00766; <0.01).

	Superficial	Complex	Deep
I	7	8	2
II	5	1	1
III	10	7	3
IV	13	18	15
V	0	6	8
Total	35	40	29

### Relationship of DBF types and lymph flow pathways with lymph nodes around the clavicle

**Figure 5:**
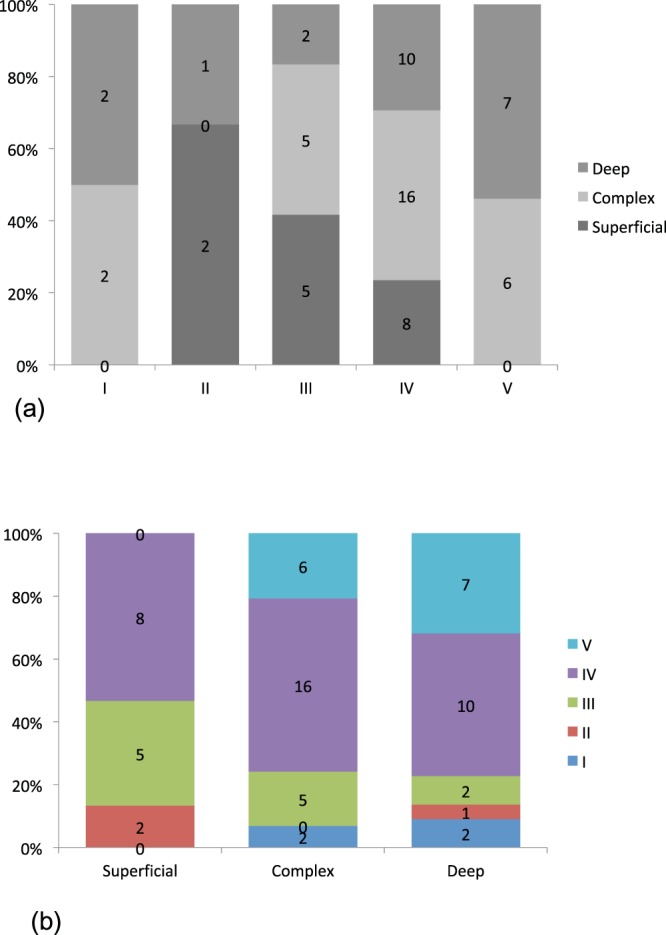
Association between the classification of dermal back flow type and lymph flow pathway in lymph node-negative cases of the affected limbs. (**a**) The superficial pathways seem to be inferior; dermal back flow appears in the distal site, similar to those cases in which lymph nodes around the clavicle were positive. It is remarkable that there were no superficial-dominant cases with a type I pattern of dermal back flow, whereas only four cases were classified into this group. At the same time, no cases (out of 13) were classified under the ‘superficial dominant’ type V group. (**b**) Type IV and V cases seem to be the most prevalent. In other words, in those cases with negative lymph nodes around the clavicle in the images, few mild cases were observed regardless of the lymph flow pathway. This is comparable with the findings of Fig. 2a.

The DBF types in each lymph flow pathway group were examined in those cases that had lymph nodes around the clavicle (Table 5, Fig. 4a,b). Statistical analysis showed a negative association between lymph flow pathways and DBF types (*p* = 0.0998). The DBF types in each lymph flow pathway group were investigated in those with no lymph nodes around the clavicle (Table 6, Fig. 5a,b). The analysis showed that the association between the lymph flow pathway and the DBF type was statistically insignificant (*p* = 0.0772).

**Table 5:** Association between the types of dermal back flow and the lymph flow pathway in the cases with visualized lymph nodes around the clavicle. Statistical analysis showed a negative association between lymph flow pathway and type of dermal back flow (Fisher’s exact test, *p* = 0.0998).

Type of lymph flow	I	II	III	IV	V
Superficial dominant	7	3	5	5	0
Complex	6	1	2	2	0
Deep dominant	0	0	1	5	1

**Table 6:** Relationship between the types of dermal back flow and the lymph flow pathway in the cases with no lymph nodes around the clavicle. The correlation between the lymph flow pathway and the type of dermal back flow was not statistically significant (Fisher’s exact test, *p* = 0.0772).

Type of lymph flow	I	II	III	IV	V
Superficial dominant	0	2	5	8	0
Complex	2	0	5	16	6
Deep dominant	2	1	2	10	7

### Lymph nodes around the clavicle and DBF pattern in each lymph flow pathway type

**Figure 6:**
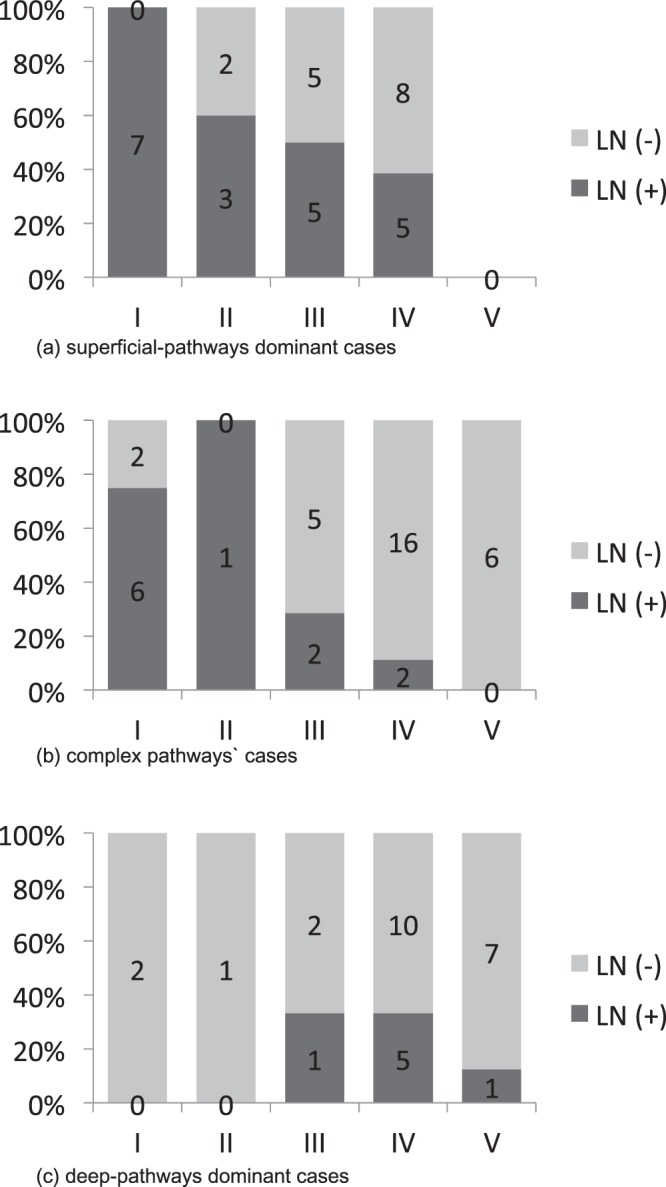
Association between the type of dermal backflow pattern and lymph nodes around the clavicle in each lymph flow pathway case. (**a**) Superficial pathway-dominant cases: All of the type I cases in this group showed lymph nodes around the clavicle, whereas no cases were of type V. There was a statistically significant association between the dermal back flow types and the lymph nodes around the clavicle (Fisher’s exact test, *p* = 0.0492). (**b**) Complex pathway cases: Most of the cases were classified as type I or II with lymph nodes around the clavicle. None of the 6 cases with type V pattern of dermal back flow showed lymph nodes, even though the superficial pathways were active based on the single photon emission computed tomography-computed tomography lymphoscintigraphy (SPECT-CT LSG) images. A statistically significant association was noted between the DBF types and the lymph nodes around the clavicle (Fisher’s exact test, *p* = 0.00151). (**c**) Deep pathways dominant cases: Most of the cases in this group did not show accumulation of the tracer around the clavicle. In fact, all the cases with type I and II patterns showed no lymph nodes around the clavicle. This finding suggests that deep lymph flow pathways rarely connect to the lymph nodes around the clavicle.

Statistical analysis showed a significant correlation between DBF types and the visualization of lymph nodes around the clavicle in superficial dominant and complex cases (superficial dominant: *p* = 0.0492, complex: *p* = 0.00151; Table 7 and b, Fig. 6a,b, Supplemental Data 1a,b). Conversely, there seemed to be a poor association in the deep dominant cases (*p* = 0.677; Table 7, Fig. 6c and Supplemental Data 1c).

**Table 7:** Association between the type of dermal backflow pattern and the lymph nodes around the clavicle in each lymph flow pathway case. Fisher’s exact test showed significant relevance in superficial dominant (*p* = 0.0492) and complex cases (*p* = 0.00151), whereas a poor association was noted in the deep dominant cases (*p* = 0.677).

	Superficial-pathway dominant cases		Complex pathway cases		Deep-pathway dominant cases	
	LN (+)	LN (−)	LN (+)	LN (−)	LN (+)	LN (−)
I	7	0	6	2	0	2
II	3	2	1	0	0	1
III	5	5	2	5	1	2
IV	5	8	2	16	5	10
V	0	0	0	6	1	7

LN (+): lymph nodes around the clavicle were observed; LN (−): no lymph nodes around the clavicle were observed.

### Laterality of the lymph nodes around the clavicle

**Figure 7:**
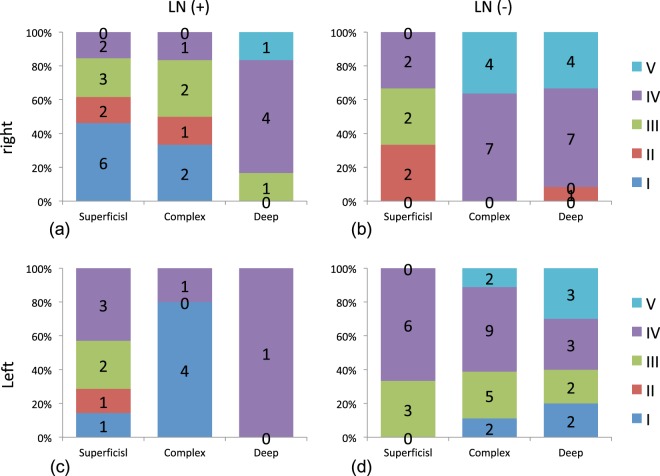
Association between the lymph flow pathway and the type of dermal black flow in the affected limbs. (**a**) Lymph nodes around the clavicle were positive in the right affected limbs (Fisher’s exact test, *p* = 0.189). (**b**) Lymph nodes around the clavicle were negative in the right affected limbs (Fisher’s exact test, *p* = 0.0401). (**c**) Lymph nodes around the clavicle were positive in the left affected limbs (Fisher’s exact test, *p* = 0.272). (**d**) Lymph nodes around the clavicle were negative in the left affected limbs (Fisher’s exact test, *p* = 0.427).

Laterality was suspected with regard to the visualization of the lymph nodes around the clavicle (*p* = 0.04178). The laterality of the DBF types (*p* = 0.258) and lymph flow pathway (*p* = 0.270) was not statistically significant (Fig. 7).

## Discussion

This study’s population was mostly female patients who had been diagnosed with breast cancer. Hence, the baseline characteristics of the patients were uniform. All DBF types in the non-affected sides were categorized as type I, similar to that in our previous study using conventional lymphoscintigraphy^10^. Therefore, there may be little difference between the reconstructed images obtained using SPECT-CT LSG and conventional lymphoscintigraphy.

On the basis of the findings of clinical examination, all 98 limbs on the non-affected side were classified as type I DBF, which demonstrated that, in general, no lymph stasis occurred in the upper limb without clinical symptoms of lymphoedema. Lymph nodes around the clavicle were found in almost all of the non-affected sides and there were no deep pathway-dominant types in cases with non-affected limbs. This finding implies that superficial pathways comprise the dominant lymph flow to the lymph nodes around the clavicle.

As the DBF appeared at the more distal part of the upper limb, the number of lymph nodes around the clavicle appeared to decrease. This is similar to a finding in our previous report on conventional lymphoscintigraphy of secondary upper limb lymphoedema^10^. In addition, when considering the possibility that the superficial pathways to the lymph nodes around the clavicle might be dominant, the deep pathways may be more dominant pathways in severe cases, in which the DBF appears in the distal portions.

Our results suggest that deep layer pathways may become dominant as the severity of the DBF pattern increases. Conversely, the number of superficial pathway dominant cases in type I DBF patterns is lower than that in type II (Fig. 3). Since the rate of superficial dominant cases seemed to decrease from type II to type V, this reversal phenomenon is unusual. The classification of the lymph flow pathways in affected limbs with type 1 DBF patterns is similar to that in the non-affected limbs, when compared with cases of type II DBF patterns, although there was no statistically significant association.

The lymph flow of the upper limb to the lymph nodes in the ipsilateral axilla is thought to be deep pathway-dominant, considering the fact that all except three cases had undergone axillary dissection. We may build a hypothesis as follows. First, the deep lymph flow pathways are affected soon after axillary dissection, following which the superficial pathways become dominant and the lymph flows to the lymph nodes around the clavicle. Second, when these alternative pathways are overloaded, the lymphoedema progresses along with histological changes in the lymphatic vessels and the surrounding tissue in the superficial layer (i.e. the superficial lymphatics become disordered). At the same time, the deep pathways, which are surrounded by muscle that rarely undergoes fibrosis, become dominant. This hypothesis is consistent with that of a previous cadaver dissection study^15^.

Four upper limbs in three patients did not undergo axillary dissection. Two patients out of three (three limbs out of four) were treated with radiotherapy; the other limb showed accumulation of the tracer in the axillary lymph nodes of the affected side, and the deep pathways were dominant. This finding supports the hypothesis that deep layer pathways mainly contribute to the lymph flow to the axillary lymph nodes.

Moreover, the superficial pathways are thought to be disordered in severe cases of lymphoedema, on the basis of the association between the DBF pattern and clinical severity. In a clinical scenario, this phenomenon explains the need for higher pressure in the compressive sleeves and increased force during massage for severe cases of lymphoedema.

There was no correlation between the types of DBF and the types of lymph flow pathways in each group classified by the accumulation of the tracer to the lymph nodes around the clavicle. This may mean that, for cases in which lymph nodes around the clavicle are positive on SPECT-CT LSG, the superficial pathways are almost dominant in each case (Supplemental Data 2a). At the same time, for the cases in which lymph nodes around the clavicle are negative on SPECT-CT LSG, the deep pathways are more functional than the superficial pathways are, regardless of the DBF type (Supplemental Data 2b).

There was no statistical correlation between the lymph nodes around the clavicle and the DBF type in the cases in which the deep pathways are dominant. This finding may indicate that the DBF type is IV or V in most lymphoedema cases in which deep pathways are dominant. In other words, severe cases are often seen in patients whose deep lymph flow pathways are dominant whether or not the lymph nodes around the clavicle are positive on the SPECT-CT LSG images.

In contrast, among cases in which superficial pathways are dominant, most are classified into a severe DBF pattern if the lymph nodes around the clavicle are negative, which is compatible with the findings shown in Fig. 6.

It was expected that there would be no statistical significance in the laterality of the association between the DBF types and the types of lymph flow pathways. However, statistical significance was observed in the laterality of the positive rate of lymph nodes around the clavicle. One of the causes of this finding is that there is a little difference in the lymph flow around the clavicle between the right and left sides; the right-sided lymph nodes drain the right side of the head, neck, and right upper limb, whereas the left-sided lymph nodes drain the other parts of the body. Reperfusion of the tracer after uptake into the blood circulation might have had some influence on these findings. Other causes should be investigated in the future.

Our study was not without limitations. First, there was little objectivity in classifying DBF types and lymph flow types. Particularly for lymph flow types, it was surprising that split decisions between two judges were observed in only two limbs. Perhaps, the precision of image recognition by artificial intelligence will improve in the future, so that studies such as ours could be reconfirmed with the help of digital indicators. Second, with respect to the number of patients enrolled, the upper limit was 101 because we were restricted in our use of 99mTc-human serum albumin because of the difficulty in obtaining transfused blood preparations in our country. Other findings would have been observed if 99mTc-phytate had been used. Third, there was little anatomical or histological evidence of the existence of deep flow pathways. In other words, we cannot prove that the deep flow pathways on the SPECT-CT LSG images indicate the lymphatic vessels. Further anatomical or histological study will be needed.

**Figure 8:**
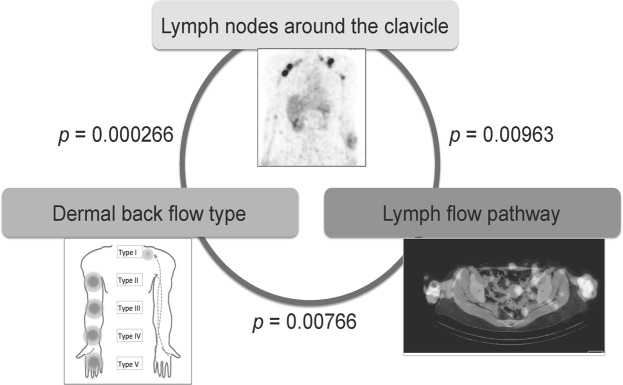
Association between the dermal back flow type, lymph flow pathway, and the lymph nodes around the clavicle on the single photon emission computed tomography-computed tomography lymphoscintigraphy images of secondary upper limb lymphoedema. Fisher’s exact test showed a statistically significant association between the dermal back flow (DBF) type and the lymph nodes around the clavicle (*p* = 0.000266), the lymph nodes around the clavicle and the lymph flow pathway (*p* = 0.00963), and the DBF type and the lymph flow pathway (*p* = 0.00766).

In conclusion, we found an association between the DBF type, lymph flow pathway and the lymph nodes around the clavicle on SPECT-CT LSG images of secondary upper limb lymphoedema (Fig. 8). According to these findings, as the severity of lymphoedema worsens, the lymph flow in the deep layer becomes dominant, and the flow into the lymph nodes around the clavicle decreases. In addition, the superficial pathways are speculated to be the main lymph flow to the lymph nodes around the clavicle. These findings may provide some insight into lymphoedema pathology as well as the anatomy and physiology of the lymphatic system. Conservative therapy such as manual lymph drainage in severe cases of lymphoedema could focus on the deep flow pathways, based on the findings of this study. Further studies that can verify these findings are warranted.

## Methods

### Ethics Statement

The use of SPECT-CT LSG images and other data of the patients was approved by the Ethics Committee of Yokohama City University Hospital (study number B110707025, B151105012) and conformed to the guidelines described in the latest revision of the Helsinki Declaration. Written informed consent for all data was obtained from the participants.

### Patient selection

All individuals included in this study were diagnosed with secondary upper limb lymphoedema on the basis of clinical history, subjective symptoms, and physical findings according to the classification established by the International Lymphology Society or by circumferential measurement. They underwent SPECT-CT LSG between November 1, 2012 and March 31, 2016. Patients who had undergone surgical treatment for lymphoedema before the imaging examinations were excluded. In total, 101 patients with lymphoedema were included, of which 5 were male and 96 were female. The mean age was 57.5 years (±10.5, range 34 to 90 years). The number of affected limbs was 104 because both upper limbs were affected in three patients. One patient with bilateral lymphoedema had breast cancer that had been treated with chemotherapy without surgery.

### SPECT-CT LSG and evaluation

All patients underwent SPECT-CT LSG according to the protocol at our hospital. For the diagnostic imaging procedure, 200 mBq/ml of the radioisotope 99mTc-human serum albumin was used. The medium was injected into the subcutaneous tissue of the interdigital spaces between the 1^st^ and the 2^nd^ fingers and between the 4^th^ and 5^th^ fingers of both hands. Two hours after injection of radioisotope, images of the upper part of the body were taken by a combined SPECT-CT system equipped with a dual-headed gamma camera, Symbia T16 (Siemens Healthcare, Erlangen, Germany). On the basis of scintigrams, the limbs were categorized into five types according to the DBF location and two subtypes on the basis of the images of the lymph nodes around the clavicle; the lymph nodes were classified as either positive or negative^10^. Next, on the basis of the SPECT-CT LSG images, the pattern of the lymph flow in each limb was classified into three types: superficial pathways in the subcutaneous tissue that were dominant as the superficial dominant type, deep pathways in the muscle layer that were thought to be dominant as the deep dominant type, and both pathways with almost equal imaging as the complex type (Supplemental Data 3–8). TM, a plastic surgeon, and AK, a medical student at the time of this study who was not familiar with lymphoedema patients or SPECT-CT LSG images, performed the assessment of the images. A final decision was made after both judges had finished evaluating the images, and split decisions were resolved by discussion between the two judges.

### Data analysis

The database of the primary data was organized with FileMaker Pro 11 Advanced (FileMaker, Inc., Santa Clara, CA, US). The primary data were compiled with Microsoft EXCEL for Mac 2011 (Microsoft Japan Co., Ltd., Tokyo, Japan).

For the statistical analysis, Fisher’s exact test was performed using R for Windows (x64 3.1.3; R Foundation for Statistical Computing, Vienna, Austria). Each factor was considered independent of the other as a null hypothesis. A *P* value of < 0.05 was considered statistically significant.

## Supplementary information

Supplemental Information

Supplemental data 3a

Supplemental data 4a

Supplemental data 5a

Supplemental data 6a

Supplemental data 7a

Supplemental data 8a

## Data Availability

The data that support the findings of this study are available from the corresponding author upon reasonable request.
