# Supplementary material for: Pathological changes in the lymphatic system of patients with secondary upper limb lymphoedema
Source: Sci Rep. 2019 Jun 11;9:8499. doi: 10.1038/s41598-019-44735-w (PMC6560042; doi:10.1038/s41598-019-44735-w)
Supplement: Supplementary file 1 — Supplemental Information [file 41598_2019_44735_MOESM1_ESM.pdf]

Title

Pathological changes in the lymphatic system of patients with secondary upper limb lymphoedema

Authors

*Taro Mikami[1], MD, PhD, Associate Professor, Department of Plastic and Reconstructive Surgery, Yokohama City University, School of Medicine
236-0004 Fukuura 3-9, Kanazawa-ku, Yokohama City, Kanagawa Prefecture, Japan
zeong3@mac.com
Phone +81-45-787-2709
FAX +81-45-783-5351

Asumi Koyama[2], MD, Resident, Tokyo Medical Center
152-8902 Higashigaoka 2-5-11, Meguro-ku, Tokyo, Japan
akoyama.917@gmail.com

Koukichi Hashimoto[1], Researcher and Data Analyst, Department of Plastic and Reconstructive Surgery, Yokohama City University
236-0004 Fukuura 3-9, Kanazawa-ku, Yokohama City, Kanagawa Prefecture, Japan
khashi39@jcom.zaq.ne.jp

Jiro Maegawa[3], MD, PhD, Professor, Department of Plastic and Reconstructive Surgery, Yokohama City University, Graduate School of Medicine
236-0004 Fukuura 3-9, Kanazawa-ku, Yokohama City, Kanagawa Prefecture, Japan
maekawaj@med.yokohama-cu.ac.jp

Yuichiro Yabuki[1], MD, Assistant Professor, Department of Plastic and Reconstructive Surgery, Yokohama City University, School of Medicine
236-0004 Fukuura 3-9, Kanazawa-ku, Yokohama City, Kanagawa Prefecture, Japan

yu1rou@hotmail.co.jp

Shintaro Kagimoto[1], MD, PhD, Assistant Professor, Department of Plastic and Reconstructive Surgery, Yokohama City University, School of Medicine
236-0004 Fukuura 3-9, Kanazawa-ku, Yokohama City, Kanagawa Prefecture, Japan
kagimoto@yokohama-cu.ac.jp

Shinya Kitayama[1], MD, Assistant Professor, Department of Plastic and Reconstructive Surgery, Yokohama City University, School of Medicine
236-0004 Fukuura 3-9, Kanazawa-ku, Yokohama City, Kanagawa Prefecture, Japan
kita-sin@rs5.so-net.ne.jp

Tomohiro Kaneta[4], MD, PhD, Associate Professor, Department of Radiology, Yokohama City University, School of Medicine
236-0004 Fukuura 3-9, Kanazawa-ku, Yokohama City, Kanagawa Prefecture, Japan
tomokaneta@gmail.com

Kazunori Yasumura[5], MD, Department of Plastic and Reconstructive Surgery, Kanagawa Children`s Medical Center
232-0066 Mutsukawa 2-138-4, Minami-ku, Yokohama City, Kanagawa Prefecture, Japan
burngdy@hotmail.com

Shinobu Matsubara[6], MD, PhD, Department of Vascular Surgery, Yokohama Minami Kyousai Hospital
236-0037 Mutsuurahigashi 1-21-1, Kanazawa-ku, Yokohama City, Kanagawa Prefecture, Japan
matsubara42@gmail.com

Toshinori Iwai[7], DDS, PhD, Assistant Professor, Department of Maxillofacial

Surgery, Yokohama City University, School of Medicine
236-0004 Fukuura 3-9, Kanazawa-ku, Yokohama City, Kanagawa Prefecture, Japan
iwai104oams@yahoo.co.jp

1. Department of Plastic and Reconstructive Surgery, Yokohama City University, School of Medicine
2. Tokyo Medical Center
3. Department of Plastic and Reconstructive Surgery, Yokohama City University, Graduate School of Medicine
4. Department of Radiology, Yokohama City University, School of Medicine
5. Department of Plastic and Reconstructive Surgery, Kanagawa Children`s Medical Center
6. Department of Vascular Surgery, Yokohama Minami Kyousai Hospital
7. Department of Maxillofacial Surgery, Yokohama City University, School of Medicine

Corresponding author
Taro Mikami, MD, PhD
Department of Plastic and Reconstructive Surgery, Yokohama City University, School of Medicine
236-0004 Fukuura 3-9, Kanazawa-ku, Yokohama City, Kanagawa Prefecture, Japan
E-mail address: zeong3@mac.com
Phone +81-45-787-2709
FAX +81-45-783-5351

Supplementary information

Supplemental data 1

Association between the type of dermal backflow pattern and the lymph nodes around the clavicle in each lymph flow pathway case

a. Ten cases out of 20 are classified under type I or II of DBF in the LN (+) column, while only 2 out of 15 are classified under type I or II in the LN (-) column.

b. Seven cases out of 11 are classified under type I or II of DBF in the LN (+) column, whereas only 2 out of 29 are classified under type I or II in the LN (-) column.

c. Six cases out of 7 are classified as type IV or V in the LN (+) column, whereas 17 out of 22 are classified under type IV or V in the LN (-) column. In other words, there are only few cases of type I or II of DBF patterns in both columns.

LN (+): lymph nodes around the clavicle were observed; LN (-): no lymph node was visualized around the clavicle

Supplemental data 2

Association between the severity and the lymph flow pattern in lymph node positive group and negative group

a: Lymph node positive group (Fisher's exact test, p = 0.00961); b: Lymph node negative group (Fisher's exact test, p = 0.233)

Supplemental data 3a

Cine single photon emission computed tomography-computed tomography lymphoscintigraphy (SPECT-CT LSG) data of a patient with secondary upper limb lymphoedema on the left.

The lymph flow pathway is categorized into 'deep dominant' because a high level of accumulation of the tracer is observed near the bones or in the muscle layer. On the other hand, little accumulation is seen around the left clavicle. The warm colors indicate higher accumulation of contrast media of the tracer of lymph while the cold colors express fewer accumulation of lymph. This color coding is adopted to the following cine images.

Supplemental data 3b

Reconstructed PA view of the SPECT-CT LSG images of the same patient.
No dermal back flow is observed both in the forearm and in the upper arm; therefore, this case is considered to be type I of dermal back flow pattern.

Supplemental data 4a
Cine SPECT-CT LSG data of a patient with secondary upper limb lymphoedema on the right.
The lymph flow pathway of this patient is considered to be ‘superficial dominant’ because the accumulation of the tracer in the subcutaneous layer is higher than in the muscle layer at any level of the right upper limb. The same condition is observed on the opposite side in the left arm.

Supplemental data 4b
The reconstructed PA view of the SPECT-CT LSG images of the same patient.
Dermal back flow is observed both in the forearm and in the upper arm; therefore, this case is considered to have type III pattern of dermal back flow.

Supplemental data 5a
Cine SPECT-CT LSG data of a patient with secondary upper limb lymphoedema on the left.
The lymph flow pathway is categorized into the ‘complex type’ because the accumulation of the tracer is observed both in the subcutaneous layer and in the muscle layer except for the DBF part. Connections between a deep pathway and a superficial pathway are observed in the left upper arm. In addition, the lymph nodes around the clavicle are negative in these images.

Supplemental data 5b
The reconstructed PA view of the SPECT-CT LSG images of the same patient of data 5a.
Dermal back flow is observed in the forearm and in the upper arm around the elbow; therefore, this case is considered as having a type III pattern of dermal back flow.

Supplemental data 6a

Cine SPECT-CT LSG data of a patient with secondary upper limb lymphoedema on the left.

The lymph flow pathway is regarded as ‘complex type’ because the accumulation of the tracer is observed both in the subcutaneous layer and in the muscle layer except for the DBF part. Connections between a deep pathway and a superficial pathway are observed in the left forearm. The intensity and the localization of the signal in the subcutaneous layer and the muscle layer are almost equal.

Supplemental data 6b

The reconstructed PA view of the SPECT-CT LSG images of the same patient of data 6a.

As dermal back flow is observed only in the forearm, this case is considered to be type IV of dermal back flow pattern. The lymph nodes around the clavicle are not visualized in this image.

Supplemental data 7a

Cine SPECT-CT LSG data of a patient with secondary upper limb lymphoedema on the right.

The lymph flow pathway of the right upper limb is classified into ‘superficial dominant’ because the accumulation of the tracer is mainly observed in subcutaneous layer. Accumulation of the tracer is also obvious around the right clavicle.

Supplemental data 7b

The reconstructed PA view of the SPECT-CT LSG images of the same patient of data 7a.

As dermal back flow is observed nowhere, this case is regarded as having a type I pattern of dermal back flow. The lymph nodes around the clavicle are visualized in this image as the opposite side.

Supplemental data 8a

Cine SPECT-CT LSG data of a patient with secondary upper limb lymphoedema on the right.

The lymph flow pathway is categorized into ‘deep dominant’ because a high level of the accumulation of the tracer is observed near the bones or in the muscle layer. A little accumulation is seen around the left clavicle.

Supplemental data 8b

The reconstructed PA view of the SPECT-CT LSG images of the same patient of data 8a.

As dermal back flow is observed in the hand and around the wrist, this case is regarded as type V pattern of dermal back flow. Fewer lymph nodes around the clavicle are visualized in this image than in the image of the opposite side.

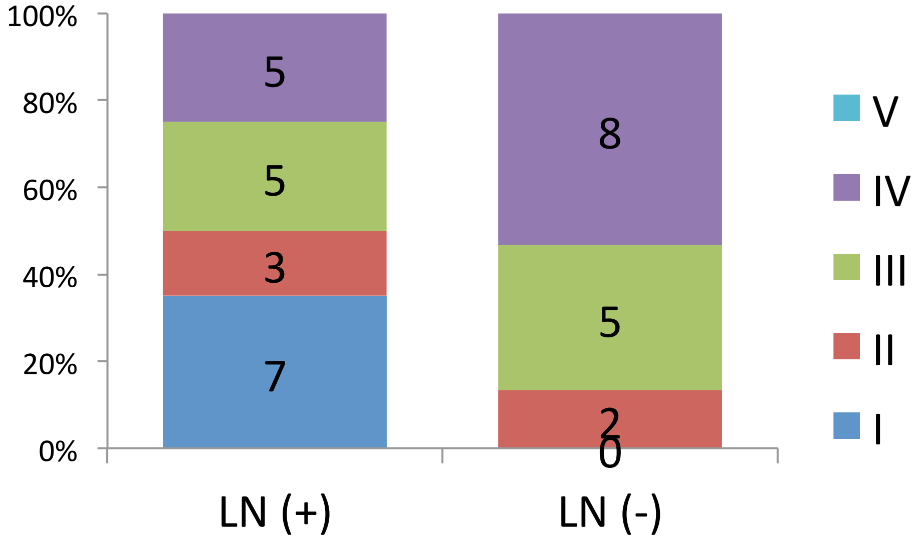

a. superficial-pathways dominant cases

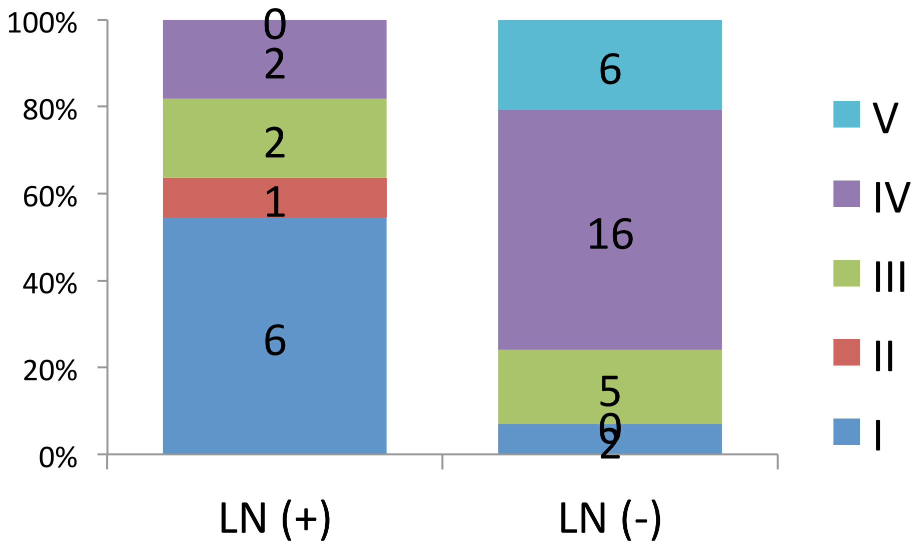

b. complex pathways` cases

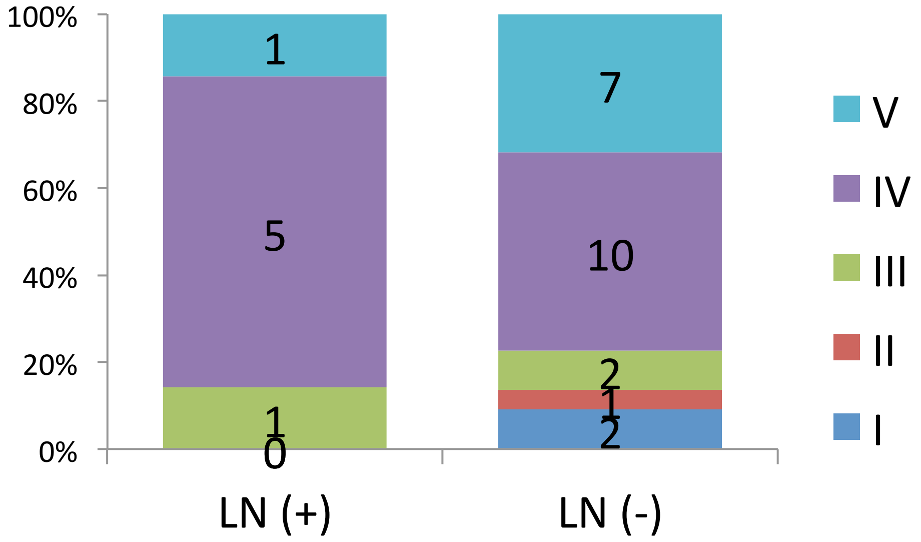

c. deep-pathways dominant cases

Supplemental data 1

Association between the type of dermal backflow pattern and the lymph nodes around the clavicle in each lymph flow pathway cases

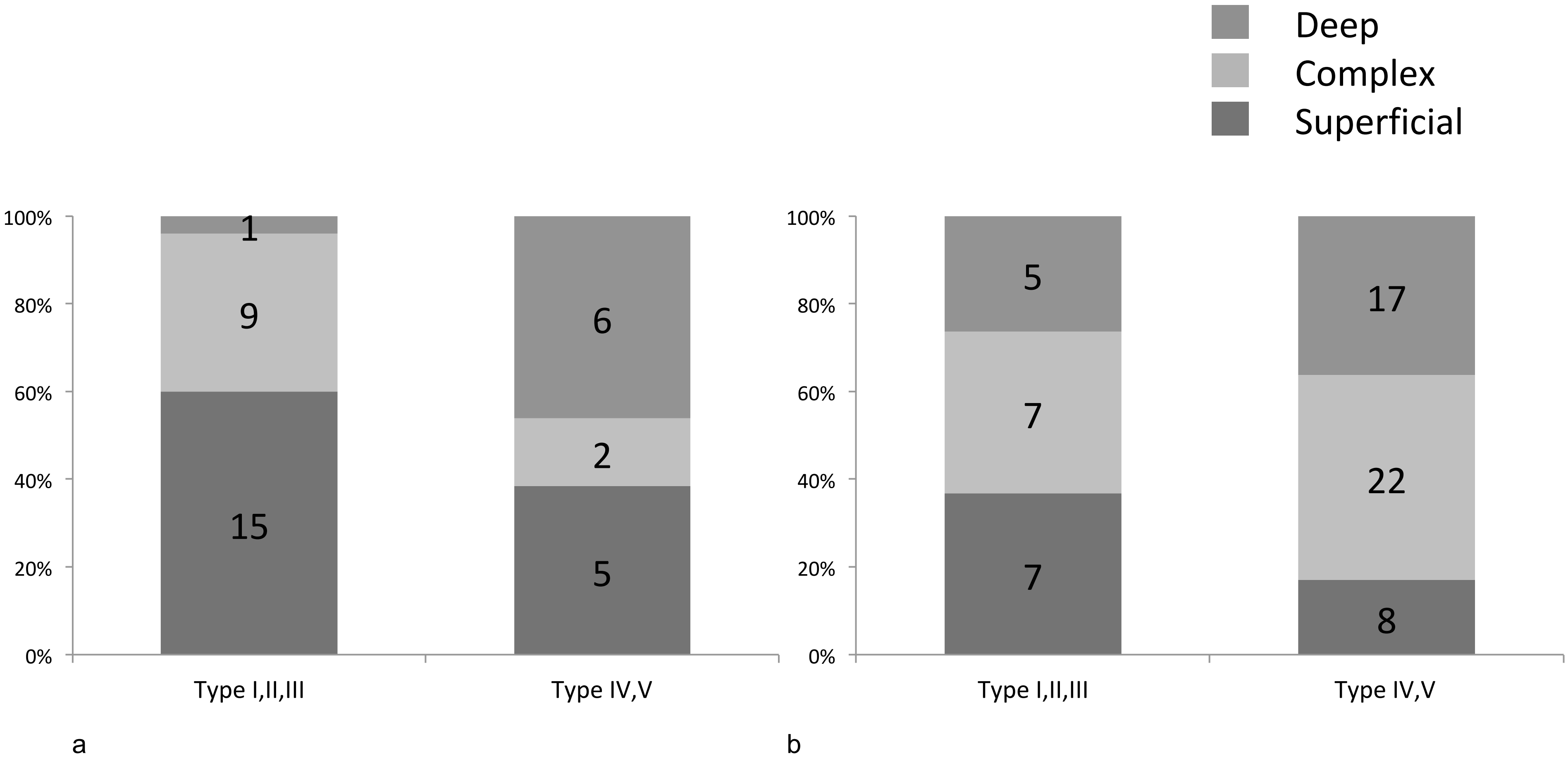

Supplemental data 2

Association between the severity and the lymph flow pattern in lymph node positive group and negative group

a: lymph node positive group (Fisher's exact test: p = 0.00961) b: lymph node negative group (Fisher's exact test: p = 0.233)

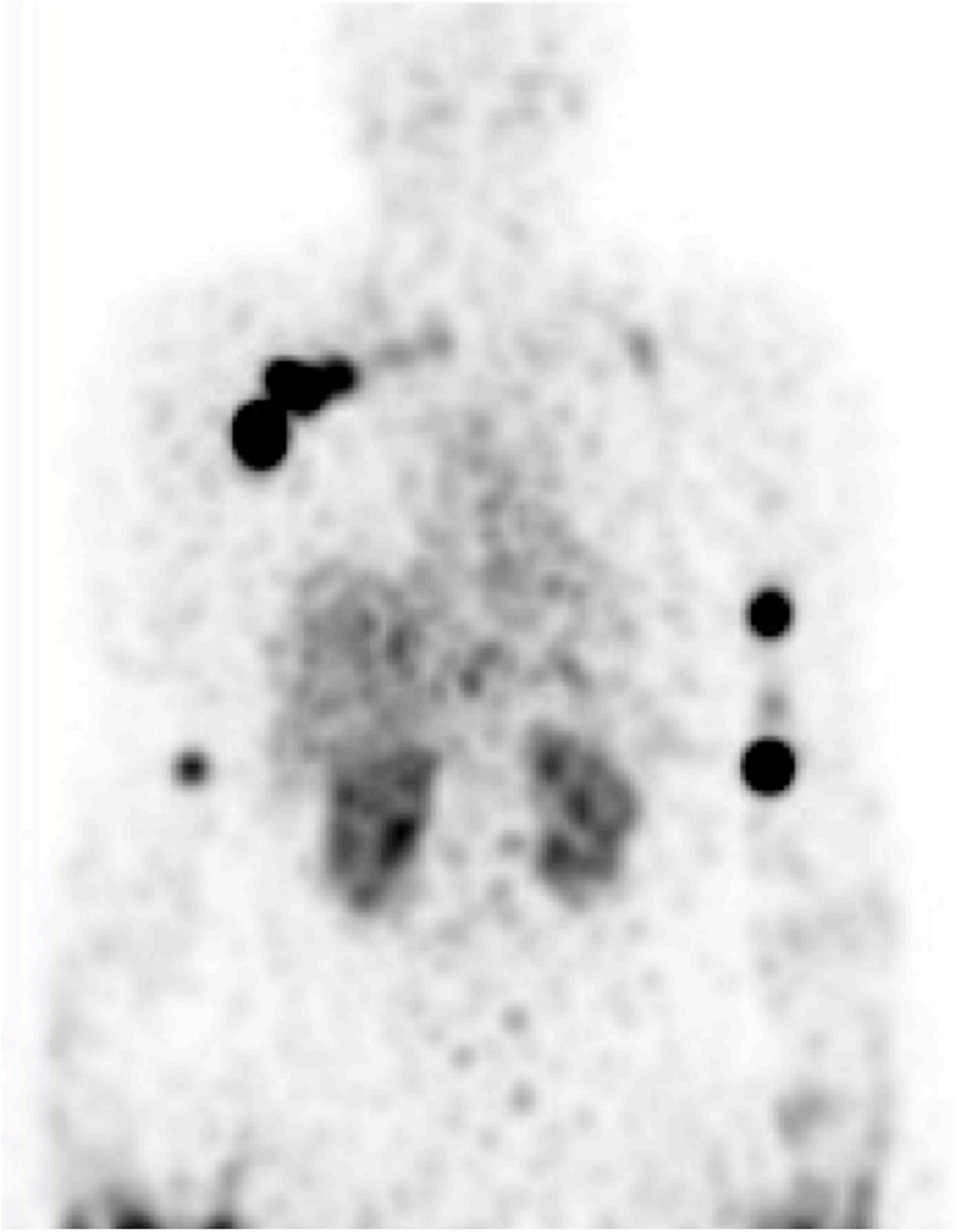

Supplemental data 3b

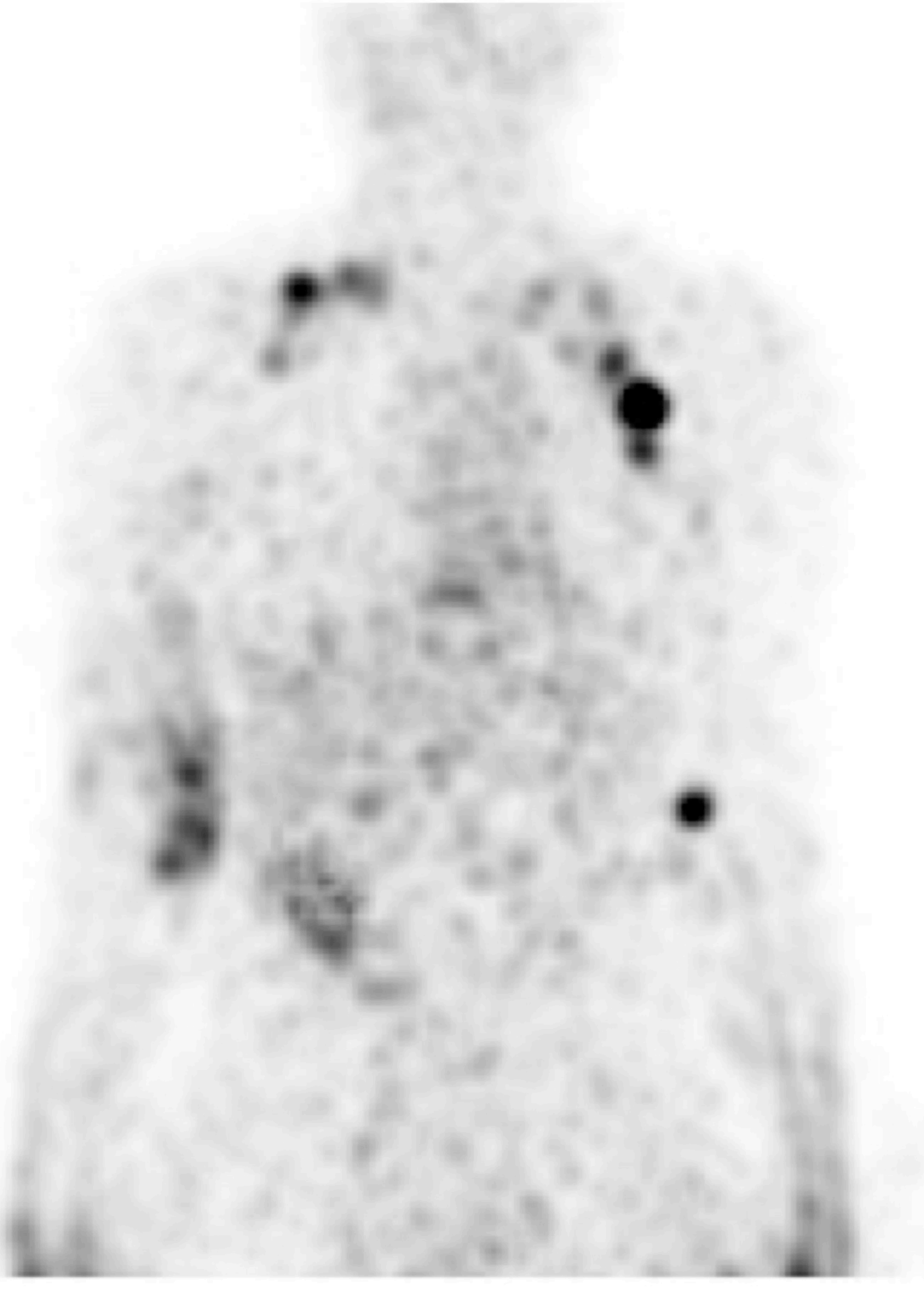

Supplemental data 4b

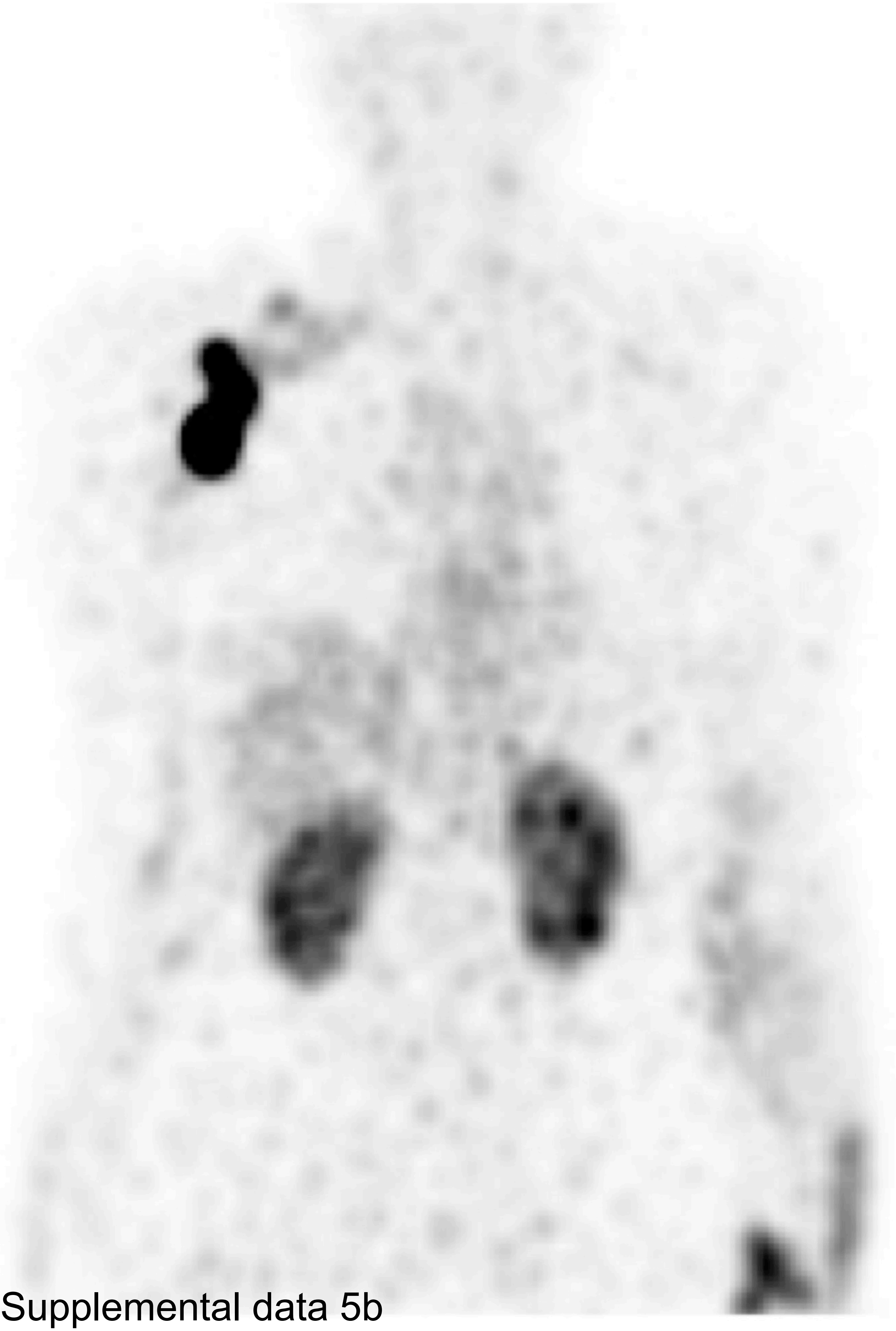

Supplemental data 5b

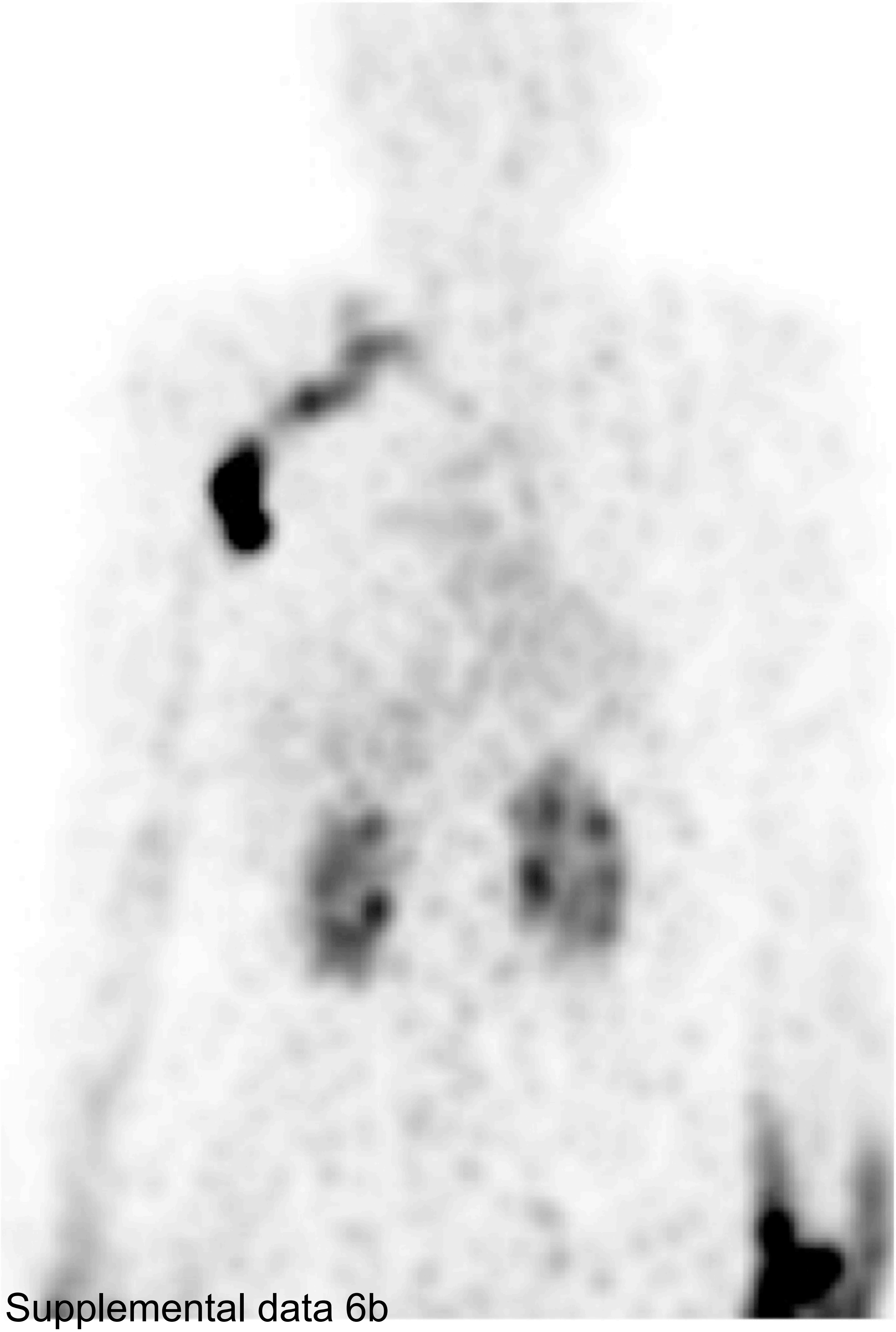

Supplemental data 6b

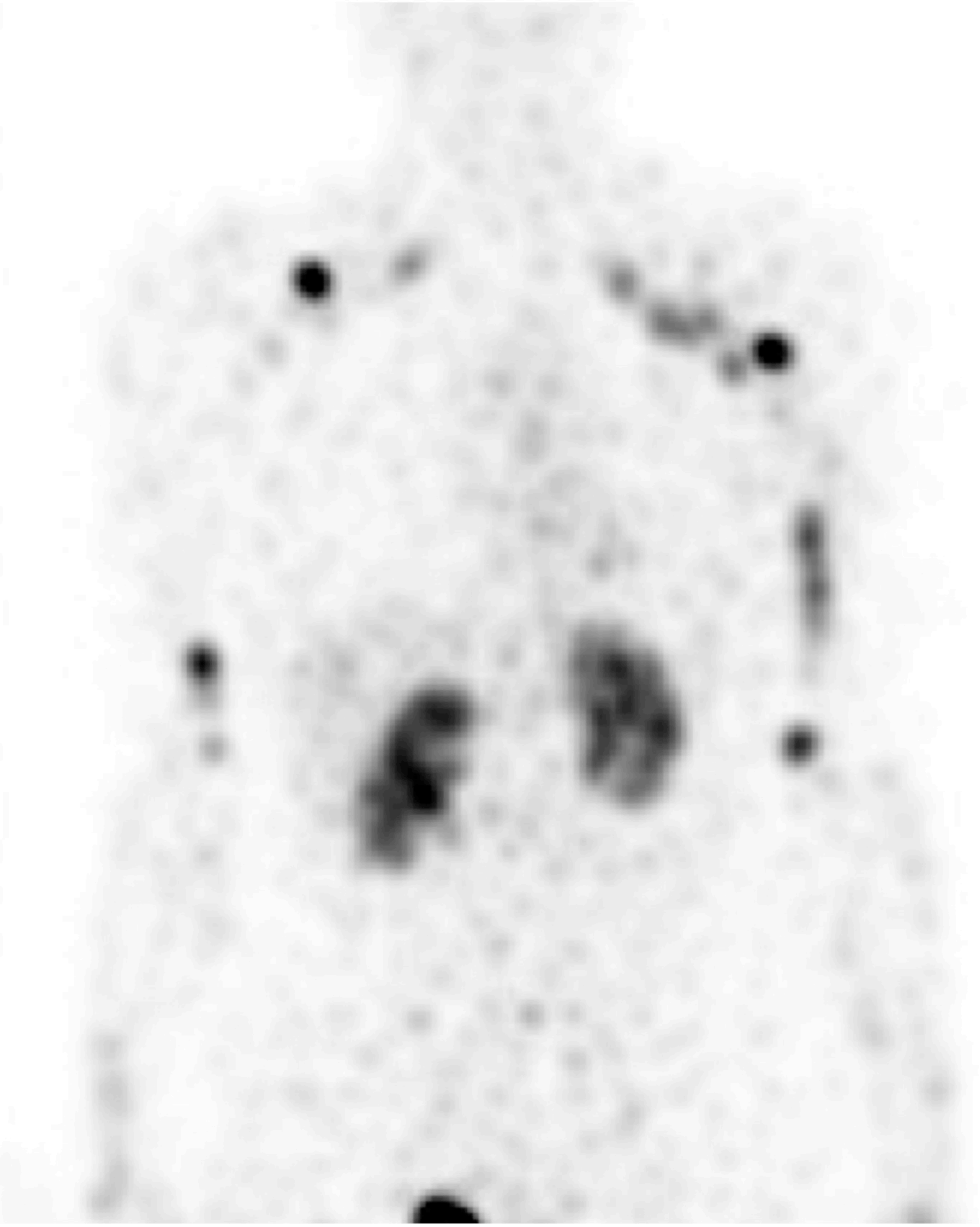

Supplemental data 7b

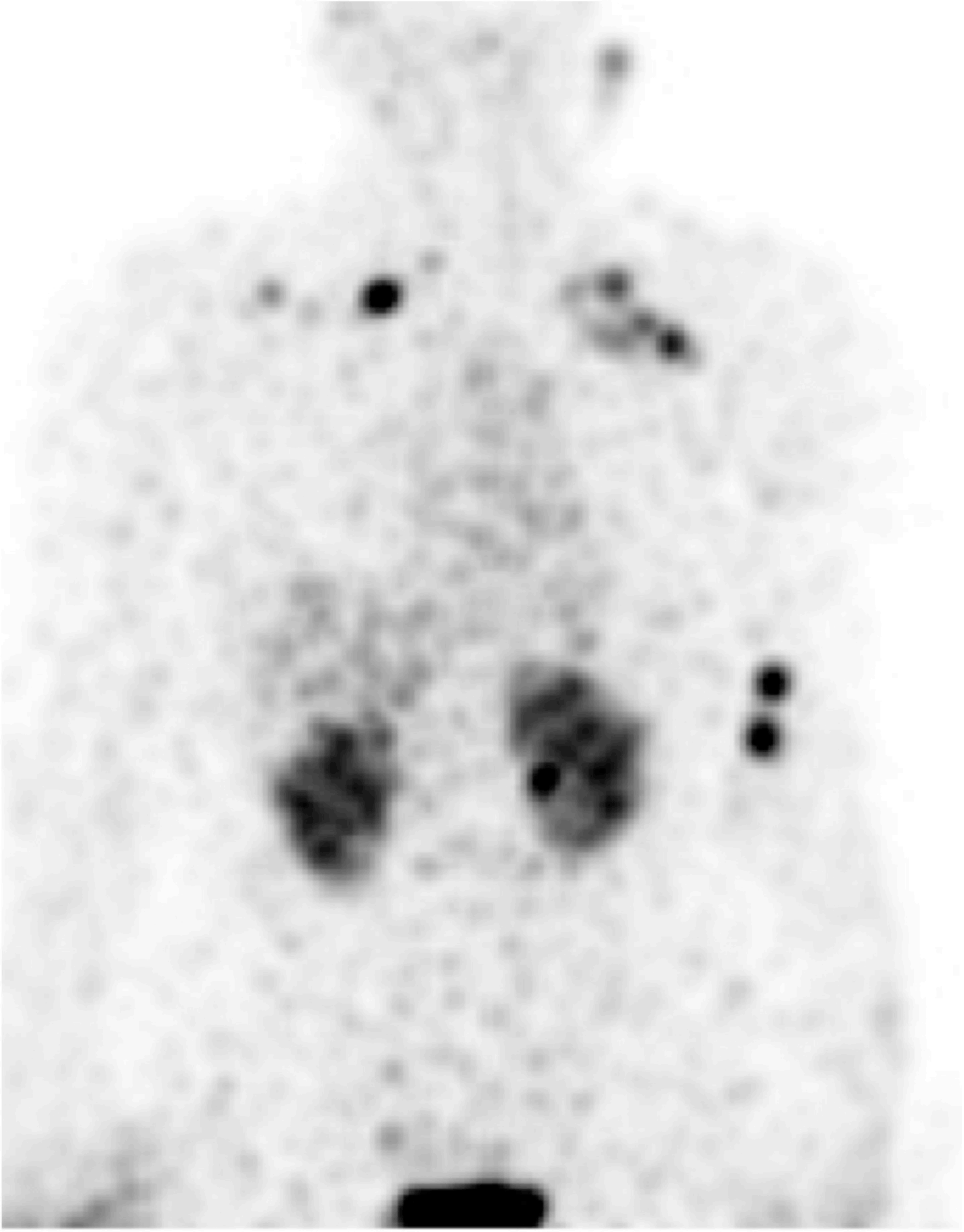

Supplemental data 8b
